# The Structure of Human Parechovirus 1 Reveals an Association of the RNA Genome with the Capsid

**DOI:** 10.1128/JVI.02346-15

**Published:** 2016-01-15

**Authors:** Sergei Kalynych, Lenka Pálková, Pavel Plevka

**Affiliations:** Central European Institute of Technology, Masaryk University, Brno, Czech Republic

## Abstract

Parechoviruses are human pathogens that cause diseases ranging from gastrointestinal disorders to encephalitis. Unlike those of most picornaviruses, parechovirus capsids are composed of only three subunits: VP0, VP1, and VP3. Here, we present the structure of a human parechovirus 1 (HPeV-1) virion determined to a resolution of 3.1 Å. We found that interactions among pentamers in the HPeV-1 capsid are mediated by the N termini of VP0s, which correspond to the capsid protein VP4 and the N-terminal part of the capsid protein VP2 of other picornaviruses. In order to facilitate delivery of the virus genome into the cytoplasm, the N termini of VP0s have to be released from contacts between pentamers and exposed at the particle surface, resulting in capsid disruption. A hydrophobic pocket, which can be targeted by capsid-binding antiviral compounds in many other picornaviruses, is not present in HPeV-1. However, we found that interactions between the HPeV-1 single-stranded RNA genome and subunits VP1 and VP3 in the virion impose a partial icosahedral ordering on the genome. The residues involved in RNA binding are conserved among all parechoviruses, suggesting a putative role of the genome in virion stability or assembly. Therefore, putative small molecules that could disrupt HPeV RNA-capsid protein interactions could be developed into antiviral inhibitors.

**IMPORTANCE** Human parechoviruses (HPeVs) are pathogens that cause diseases ranging from respiratory and gastrointestinal disorders to encephalitis. Recently, there have been outbreaks of HPeV infections in Western Europe and North America. We present the first atomic structure of parechovirus HPeV-1 determined by X-ray crystallography. The structure explains why HPeVs cannot be targeted by antiviral compounds that are effective against other picornaviruses. Furthermore, we found that the interactions of the HPeV-1 genome with the capsid resulted in a partial icosahedral ordering of the genome. The residues involved in RNA binding are conserved among all parechoviruses, suggesting an evolutionarily fixed role of the genome in virion assembly. Therefore, putative small molecules disrupting HPeV RNA-capsid protein interactions could be developed into antiviral inhibitors.

## INTRODUCTION

Human parechoviruses (HPeVs) belong to the family Picornaviridae, which contains many vertebrate and human pathogens. Parechoviruses mainly cause mild gastrointestinal diseases in neonates and young children (1–4). Occasionally, however, parechovirus infections progress to serious and debilitating illnesses, including pneumonia, flaccid paralysis, encephalitis, sepsis, and meningitis (5–7). After their discovery (8, 9), HPeVs were placed in the same genus as human enteroviruses, exemplified by polioviruses and rhinoviruses (10). In the early 1990s, a separate genus, Parechovirus, was designated, which now includes HPeVs and Ljungan viruses (11). Parechoviruses exhibit high genetic variability, with at least 16 different types (12, 13). HPeV-1, HPeV-3, and HPeV-6 are the most prevalent clinically diagnosed types (14, 15).

HPeVs are small, nonenveloped icosahedral viruses. Their virions have an outer diameter of about 300 Å and contain a positive-sense single-stranded RNA (ssRNA) genome approximately 7,500 nucleotides long (16). HPeV capsids are built out of 60 copies of each of three viral proteins, VP0, VP1, and VP3. These three capsid proteins are co- and posttranslationally cleaved from a single polyprotein and constitute a protomer—the elementary building block of the capsid. In most picornaviruses, the capsid protein VP0 is cleaved into fragments VP2 and VP4 after virion assembly (17). The RNA genome has been proposed to have a role in cleavage (18). However, in parechoviruses, VP0 proteins remain intact, even in the mature virions (11, 19).

A distinctive feature of the capsid surface of many picornaviruses is a depression encircling the icosahedral 5-fold axes, called the “canyon” (20). For many picornaviruses, the canyon is the binding site of receptors with an immunoglobulin fold (21–23). Integrins α_V_β_3_ and α_V_β_6_ were proposed to be receptors for HPeVs that possess the integrin recognition sequence arginine-glycine-aspartate (RGD) in the C terminus of VP1, including HPEV-1, -2,- 4, -5, and -6 (24, 25). The binding of receptors within the canyon induces the release of a pocket factor from a hydrophobic pocket immediately below the surface of the canyon (26). The shape of the electron density and the hydrophobic environment of the pocket suggest that the pocket factor is a lipid (27–30). The expulsion of the pocket factor is associated with a decrease in virion stability and leads to genome release.

Genome release from picornavirus virions requires structural rearrangements of the capsid (31, 32). In enteroviruses, the capsid proteins change their positions relative to each other, resulting in the opening of pores at icosahedral 2-fold symmetry axes (33–35). VP1 N termini become exposed at the particle surface, and VP4s are released from the virion. Finally, the genomic RNA leaves the empty capsids. The standard *in vitro* procedure to induce the genome release of picornaviruses by heating to 56°C (34) results in disruption of the HPeV-1 virions to pentamers (36). Thus, the *in vitro* experiments provide putative evidence that the genome release mechanism of parechoviruses might be different from that of the other enteroviruses.

Pocket-binding antipicornavirus compounds have been developed that overstabilize the capsids and thus prevent genome release (30, 37–40). The capsid-binding inhibitors targeting rhinoviruses demonstrated a moderate level of success in human clinical trials (41, 42); however, they were not effective against parechovirus infections (43).

Single-particle cryo-electron microscopy reconstructions of HPeV-1 and its complex with the integrin receptor were reported previously at a resolution of 8.5 Å (25). However, despite the impact of HPeVs on human health, the atomic-level structural details of their virions are unknown. Here, we report the crystal structure of the virion of HPeV-1 (strain Harris) determined to a resolution of 3.1 Å. We show that specific interactions of the RNA genome with the capsid proteins result in a partial icosahedral ordering of the genome. This indicates a possible role of the genome in the stability or assembly of the HPeV-1 virion.

## MATERIALS AND METHODS

### Virus preparation.

Human parechovirus (strain Harris; ATTC VR-52) was propagated in A549 human lung carcinoma cells (ATCC CCL-185). For a typical preparation, fifty 140-cm^2^ tissue culture plates were infected with HPeV-1 at a multiplicity of infection (MOI) of 0.1 at 90% confluence, and the infection was allowed to proceed for 72 h at 37°C, at which point 90% of the cells exhibited cytopathic effects. The supernatant was harvested, and any remaining attached cells were removed from the plates using cell scrapers. The supernatant was centrifuged at 7,500 × *g* for 15 min, and the resulting pellet was resuspended in 10 ml of resuspension buffer (0.25 M HEPES-HCl, pH 7.5, 0.25 M NaCl). This fraction was subjected to three rounds of freeze-thawing by sequential transfer between −80°C and 37°C and homogenized with a Dounce tissue grinder to lyse the remaining cells. Cell debris was separated from the supernatant by low-speed centrifugation at 7,500 × *g* for 15 min. The resulting supernatant was combined with that obtained during the first low-speed centrifugation step. Viral particles were precipitated by adding polyethylene glycol (PEG) 8000 and NaCl to final concentrations of 15% (wt/vol) and 0.5 M, respectively, and incubating at 4°C with mild shaking (60 rpm) overnight. The following day, the solution was spun down at 10,000 × *g* for 20 min, and the visible white precipitate was resuspended in 12 ml of the resuspension buffer. MgCl_2_ was added to a final concentration of 5 mM, and the sample was subjected to trypsin (80 μg/ml), DNase (10 μg/ml), and RNase (10 μg/ml) treatment for 30 min at 22°C. EDTA (pH 9.5) was added to a final concentration of 15 mM, and a nonionic detergent, Nonidet P-40 (Sigma-Aldrich Inc.), was added to a final concentration of 1%. The solution was incubated for an additional 20 min at 22°C and centrifuged at 3,500 × *g*, and the supernatant was spun down through a 30% (wt/wt) sucrose cushion in 30 mM Tris-HCl, pH 8.0, 250 mM NaCl at 200,000 × *g* using a Ti50.2 rotor (Beckman Coulter). The pellet was resuspended in approximately 1 ml of cold resuspension buffer and added to 10 ml of 60% (wt/wt) CsCl solution in an ultracentrifuge tube. Gradient ultracentrifugation was allowed to proceed for at least 12 h at 100,000 × *g* in a SW40Ti rotor (Beckman Coulter). The opaque virus band was extracted with an 18-gauge needle on a 3-ml disposable syringe. The virus was transferred to the resuspension buffer by multiple rounds of centrifugation using a centrifugal-filter device with a 100-kDa molecular mass cutoff (Centricon, Millipore Inc.). The yield was approximately 100 μg of purified virus.

### Crystallization, data collection, and data processing.

Purified HPeV-1 at a concentration of 3.5 mg/ml was subjected to sparse-matrix screening using a number of commercially available crystallization screens. The initial hits were obtained at room temperature using 0.1 M Tris-HCl, pH 8.0, 1 M ammonium sulfate in a sitting-drop format. The crystals measured about 30 μm in the largest dimension and diffracted X rays to a resolution of 3.1 Å in the Diamond Light Source beamline I23. In an effort to obtain larger crystals, optimization of the original crystallization conditions centered around testing various salts as precipitants and various divalent cations as additives. The best crystals were obtained in 0.1 M Tris-HCl, pH 8.0, 0.6 M ammonium sulfate, 0.1 M MgCl_2_, 5% (wt/vol) glycerol with growth at room temperature for about 3 weeks in a 2.0-μl hanging drop consisting of 0.5 μl reservoir solution and 1.5 μl of purified virus at a concentration of 3.5 mg/ml. Two independent data sets from two different crystals were collected in the Synchrotron Soleil Proxima 1 beamline and processed to a resolution of 3.1 Å using the XDS software package in space group P6_3_22 (44). The two data sets were scaled together using the program Aimless from the CCP4 suite (45, 46).

### Phasing, model building, and refinement.

HPeV-1 crystallized in space group P6_3_22. Plots of the 2-fold, 3-fold, and 5-fold self-rotation function calculated using the program GLRF had shown that virions crystallized with one of the icosahedral 3-fold axes aligned with the crystallographic 3-fold axis and icosahedral 2-fold axes aligned with the crystallographic 2-fold axes (47). Reflections between 5 Å and 3.8 Å were used for the calculations. The radius of integration was set to 140 Å. Thus, icosahedral symmetry had to be rotated (φ = 90°, φ = 90°, κ = 60°) according to the XYK polar-angle convention relative to the standard icosahedral orientation as defined by Rossmann and Blow (48). One sixth of a virus particle occupied a crystallographic asymmetric unit. The only possible way to place the particle center in the crystallographic unit cell was at the intersection of the crystallographic 3-fold and 2-fold axes [*x* = *a*/2, *y* = (*a*tg30°), *z* = *c*/4] (Table 1).

A Protein Data Bank [PDB] model of bovine enterovirus 1 (BeV1) (PDB code 1BEV) converted to polyalanine was used for molecular replacement (28). The model was rotated and positioned in the unit cell and used to calculate the initial phases for reflections up to a resolution of 10 Å in the program CNS (49, 50). The phases were refined by 15 cycles of real-space electron density averaging in the program AVE, using 10-fold noncrystallographic symmetry (NCS) (51). A mask for electron density averaging was generated using the program Mama from the Uppsala Software Factory program package by including all voxels within 5 Å of any atoms of the PDB model of the BeV1 icosahedral asymmetric unit (52). Phase extension was applied in order to obtain phases for higher-resolution reflections (53). The addition of a small fraction of higher-resolution data (one index along the *a* axis at a time) was followed by three cycles of averaging. This procedure was repeated until phases were obtained for all the reflections up to a resolution of 3.1 Å. The program SigmaA from the CCP4 package was used to include weights in the electron density calculations (45, 54). Inspection of the resulting electron density map indicated that the molecular-averaging mask was too large. Thus, a new mask was prepared based on a correlation map calculated by comparing electron density distributions among the 10 NCS-related icosahedral asymmetric units. The correlation map was calculated using the program Coma from Uppsala Software Factory (55). A cutoff value of 0.7 was used for the inclusion of voxels in the mask. The phase extension procedure was repeated using the new mask. The resulting map was of sufficient quality to enable model building. The program Buccaneer was used for automated building of the protein part of the model, utilizing the 10-fold NCS present in the crystallographic asymmetric unit (56, 57). The program Nautilus from the CCP4 suite (45) was used for the automated building of RNA chains and was able to build 3 nucleotides, while the remaining 3 nucleotides were built manually using the program Coot (58). The combined protein-RNA model from the automated building was about 80% complete with the assigned amino acid sequence. This initial model was subjected to manual rebuilding using the programs Coot and O (59) and to coordinate and B-factor refinement using the program CNS (49, 50). No water molecules were added, due to the limited resolution of the diffraction data. All the measured reflections were used in the refinement.

### Data analysis.

The volumes of the particles were calculated using the programs Mama and Voidoo from Uppsala Software Factory (60). Average radii of virions were calculated using the program Moleman2 from Uppsala Software Factory (55). Multiple-sequence alignments were carried out using the ClustalW server (http://www.ebi.ac.uk/Tools/msa/clustalw2/) (61). Figures were generated using the programs UCSF Chimera (62), PyMOL (PyMOL Molecular Graphics System, version 1.7.4; Schrödinger, LLC), and RiverM (63). Structure-based pairwise alignments of biological protomers of various picornaviruses were prepared using the program Gr-Align (64). The similarity score provided by Gr-Align was used as an evolutionary distance to construct a nexus format matrix file, which was converted into the phylogenetic tree and visualized with the program SplitsTree (65).

### Protein structure accession number.

The HPeV-1 model, structure factor amplitudes, and phases derived by phase extension have been deposited in the Protein Data Bank with PDB code 4Z92.

## RESULTS AND DISCUSSION

### Quality of diffraction data and HPeV-1 structure.

The structure of HPeV-1 was determined by X-ray crystallography at a resolution of 3.1 Å. The electron density map resulting from 10-fold NCS averaging enabled the HPeV-1 capsid proteins to be built, except for residues 1 to 31 and 289 of VP0, 1 to 24 and 217 to 235 of VP1, and 1 to 14 of VP3. Identifying the sequences of the individual residues was straightforward, due to the good quality of the electron density map. Six nucleotides corresponding to the RNA genome were modeled per icosahedral asymmetric unit. Water molecules could not be modeled because of the limited resolution of the available diffraction data. If calculated, the *R*_free_ value would have been very close to the *R*_work_ value due to the 10-fold NCS (66). Thus, all measured reflections were used in the crystallographic refinement. The basic crystallographic structure quality indicators are listed in Table 1.

**TABLE 1:** Diffraction data and structure quality indicators

Parameter	Value
Space group	P6_3_22
Cell parameters	
*a*, *b*, *c* (Å)	399.5, 399.5, 332.9
α, β, γ (°)	90, 90, 120
Resolution shell^*a*^	65.0–3.1 (3.15–3.10)
No. of observations^*a*^	1,187,478 (5,621)
No. of unique reflections^*a*^	233,083 (4,306)
Observation multiplicity^*a*^	5.4 (1.3)
Completeness^*a*^	78.2 (31.8)
*R*_merge_ (%)^*a*^,^*b*^	0.354 (0.904)
*I*/σ(*I*)^*a*^	5.0 (0.9)
*R*_factor_^*a*^	0.29 (0.41)
No. of protein atoms^*c*^	5,283
No. of RNA atoms^*c*^	119
Average B factor protein (Å^2^)	50
Average B factor RNA (Å^2^)	71
Ramachandran plot statistics	
Preferred regions (%)^*d*^	90
Allowed regions (%)^*d*^	9.41
Disallowed regions (%)^*d*^	0.59
RMSD^*e*^ bond angles (°)	0.005
RMSD bond lengths (Å)	1.16

a Statistics for the highest-resolution shell are shown in parentheses.

b *R*_merge_ = Σ_*h*_Σ_j_|*l_hj_* − <*l_h_*>|/ΣΣ|*l_hj_*|.

c Statistics are given for one icosahedral asymmetric unit.

d As calculated by Molprobity (79).

e RMSD, root mean square deviation.

### Structures of HPeV-1 capsid proteins and virion.

**FIG 1:**
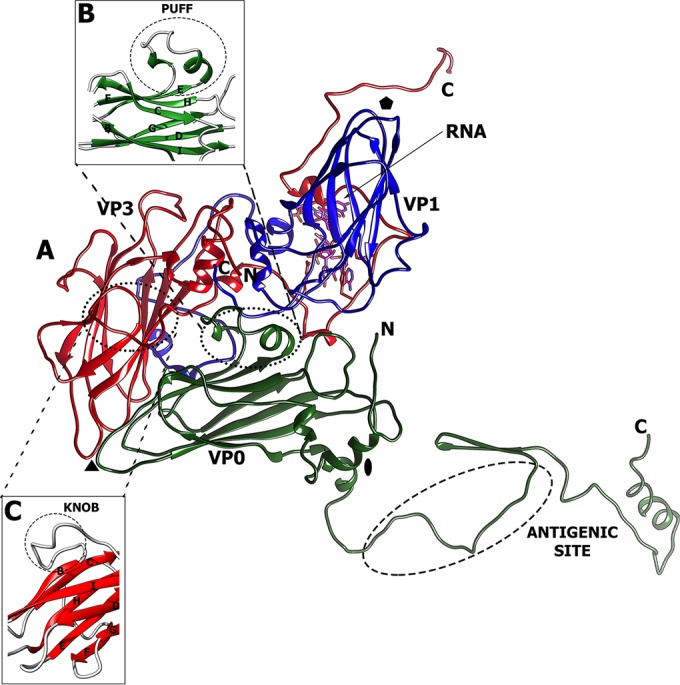
Icosahedral asymmetric unit of HPeV-1. (A) Cartoon representation of major capsid proteins: VP0 (green), VP1 (blue), and VP3 (red). The locations of the 5-fold, 3-fold, and 2-fold symmetry axes are indicated by a pentagon, a triangle, and an oval, respectively. (B and C) The puff (B) and knob (C) loops constituting the major capsid surface features.

The icosahedral asymmetric unit of HPeV-1 consists of subunits VP0, VP1, and VP3 (Fig. 1). The core of each of the capsid proteins is a jelly roll β-sandwich composed of two β-sheets, each containing four antiparallel β-strands. The β-strands are conventionally named B to I, and the two β-sheets contain strands BIDG and CHEF, respectively. The C termini of the capsid proteins are located on the virion surface, while the extended N termini mediate interactions among the capsid proteins and with the RNA genome on the inner surface of the capsid.

**FIG 2:**
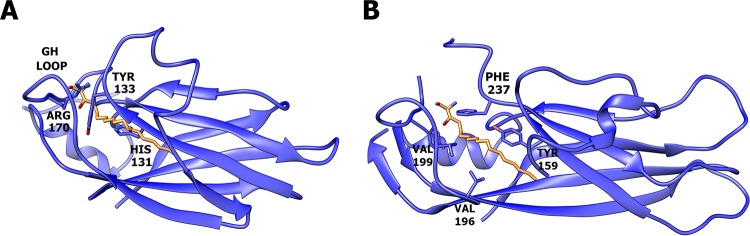
VP1 of HPeV-1 does not contain a hydrophobic pocket. The VP1 proteins of HPeV-1 (A) and poliovirus type 1 (B) are shown as cartoon representations. The pocket factor in poliovirus type 1 is shown as a stick model in orange. The side chains of residues that interact with the pocket factor are also shown as sticks. In panel A, the poliovirus type 1 pocket factor was superimposed onto the HPeV-1 structure. However, the pocket is not formed, and the side chains of several residues clash with the pocket factor.

A pocket in the capsid protein VP1, which is present in most picornaviruses, can be targeted by capsid-binding inhibitors. This pocket is not formed in HPeV-1 (Fig. 2). Bulky amino acid side chains of His-131, Tyr-133, and Arg-170 fill the equivalent volume of the cavity, and the mouth of the pocket is occluded by the main-chain atoms of the GH loop (Fig. 2A). This explains the previous findings that the capsid-binding inhibitors that inhibit enteroviruses and rhinoviruses are not effective against parechoviruses (43).

**FIG 3:**
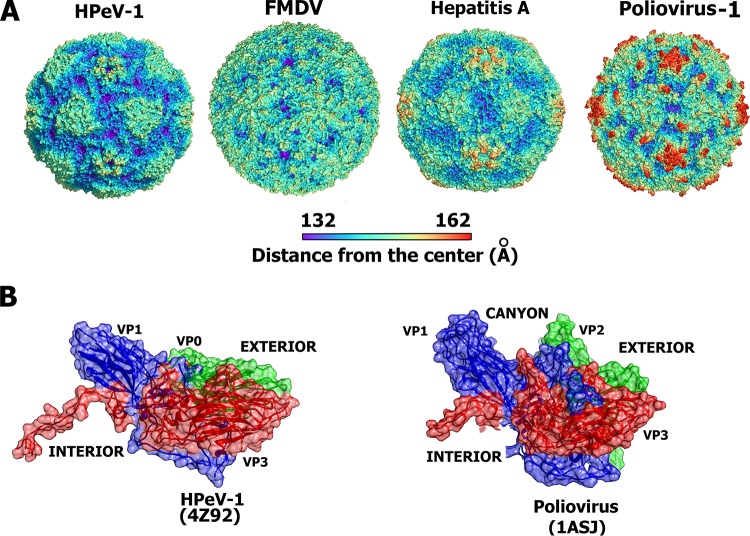
Comparison of virion surface features of selected picornaviruses. (A) Molecular surfaces of selected picornaviruses rainbow colored according to the distance from the particle center. (B) Comparison of side views of biological protomers of HPeV-1 and poliovirus type 1 showing that the canyon is not formed in the HPeV-1 structure. The subunits VP0 and VP2 are shown in green, VP1 in blue, and VP3 in red.

Of the picornaviruses that have been structurally characterized, HPeV-1 has the smallest observed virion, with an average diameter of 247 Å (Table 2). The surface of the HPeV-1 virion is relatively flat in comparison to the other picornaviruses because no canyon is formed (Fig. 3A). This is due to the relative shortening of features on the capsid surface that form the borders of the canyon. The HI loop of VP1, which forms protrusions around a 5-fold axis, is shortened from 12 residues in poliovirus type 1 (PDB code 1ASJ) to 6 residues in HPeV-1, resulting in a decrease in the height of the “northern rim” of the canyon. The “southern rim” of the canyon is mostly formed by two loops called the “puff” and the “knob.” The puff is a loop between βE and βF of VP0/VP2 and in HPeV-1 contains two short 3._10_ helices connected by a 10-residue loop (Fig. 1B). The knob is a loop preceding βB of VP3 and in HPeV-1 contains 10 residues (Fig. 1C). The knob and puff of HPeV-1 are 2 and 65 residues shorter than those found in poliovirus type 1. In addition, the residues forming the puff and knob do not protrude away from the viral surface, as observed in poliovirus type 1 (Fig. 3B).

**TABLE 2:** Physical parameters of selected picornaviruses

Virus^*a*^/PDB code	Diameter (Å)^*b*^	Virion vol (10^6^ Å^3^)	Genome size (nucleotides)	RNA density (Å^3^/nucleotide)
HPeV-1	247	6.88	7,321	10.6
Poliovirus type 1/1ASJ	256	8.70	7,433	8.6
HRV-16/1AYM	258	8.44	7,124	8.4
BeV1/1BEV	259	8.14	7,414	8.0
FMDV/1BTT	258	7.19	8,176	11.3
HAV/4QPI	254	7.45	7,478	10.0

a HRV-16, human rhinovirus type 16.

b Determined as the distance between the center of mass of the capsid protein protomer and the center of the virion.

**FIG 4:**
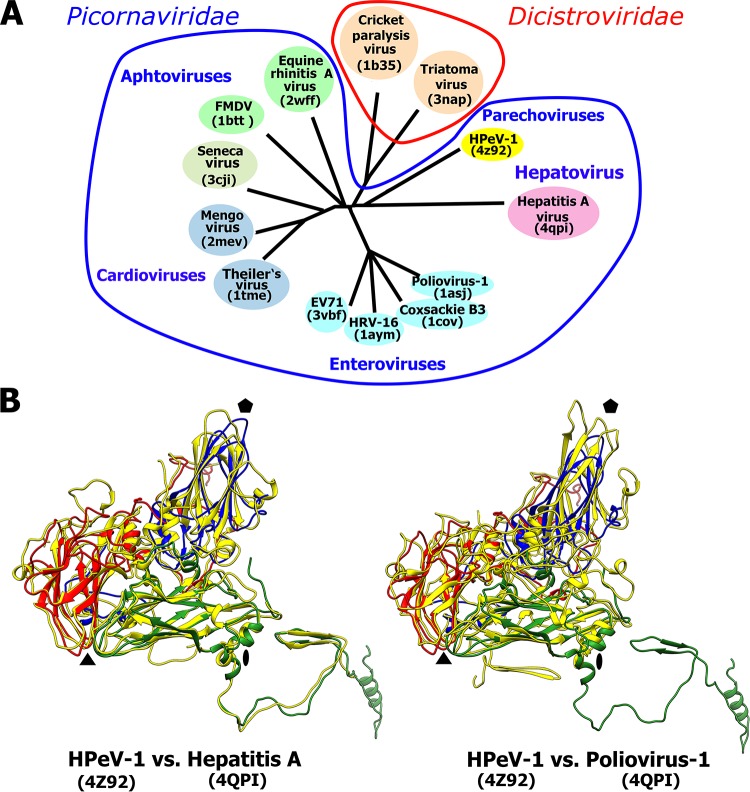
Evolutionary relationship among viruses from the families Picornaviridae and Dicistroviridae based on structural alignment of capsid proteins. (A) Phylogenetic tree based on structural similarity of icosahedral asymmetric units of the indicated viruses. For details on the construction of the diagram, see Materials and Methods. (B) Icosahedral asymmetric unit of HPeV-1 (VP0, green; VP1, blue; VP3, red) superimposed on those of HAV and poliovirus type 1 (all capsid proteins, yellow). Pentagons, triangles, and ovals indicate the positions of the icosahedral 5-fold, 3-fold, and 2-fold symmetry axes, respectively.

The availability of the HPeV-1 virion structure enabled the construction of a structure-based phylogenetic tree comparing HPeV-1 to 12 other viruses from the order Picornavirales (Fig. 4A). The phylogenetic analysis shows that the HPeV-1 capsid is most similar to that of hepatitis A virus (HAV) from the family Picornaviridae and to those of cricket paralysis virus and triatoma virus from the family Dicistroviridae (Fig. 4A). The relatively close evolutionary relationship between HPeV-1 and HAV is further indicated by the similar positions of the structured N-terminal arms of HPeV-1 VP0 and of HAV VP2 (Fig. 4B). The structured parts of the N termini of the two viruses mediate interactions among the capsid protein protomers along a line connecting the icosahedral 2-fold and 3-fold axes of the capsid (Fig. 4B). The N-terminal arm of VP2 of poliovirus type 1 has the same function; however, in poliovirus type 1, the structured part of the N-terminal arm of VP2 mediates interactions among protomers different than those in the virions of HPeV-1 and HAV (Fig. 4B). This is an example of domain swapping where part of a protein retains its function; however, its location in the quaternary complex is different. The closer similarity of the HPeV-1 capsid to those of viruses from the family Dicistroviridae than to those of viruses from the family Picornaviridae, with the exception of HAV, might be due to the differences in the processing of the polyprotein precursor of capsid proteins. The amino acid sequence of the VP4 subunit is located between VP2 and VP3 in viruses from the family Dicistroviridae while it is located before the VP2 sequence in viruses from the family Picornaviridae. In contrast, parechovirus capsids do not contain VP4, and this might be reflected in the structural organization of the capsid. Thus, HPeVs might represent an evolutionary link between picornaviruses and other virus families in the order Picornavirales, as was previously suggested for HAV (67).

### Integrin receptor binding site of HPeV-1.

**FIG 5:**
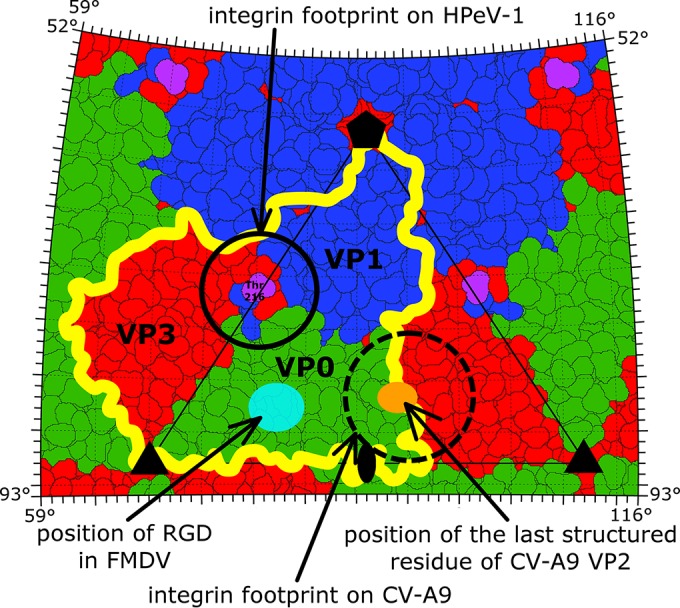
Comparison of α_V_β_6_ integrin receptor binding sites of HPeV-1, FMDV, and CV-A9. Shown is a stereographic projection of HPeV-1 surface residues, with subunits VP0, VP1, and VP3 in green, blue, and red, respectively. The yellow line shows the border of a selected protomer. Features of FMDV and CV-A9 were plotted onto the HPeV-1 surface. The last structured C-terminal residue of HPeV-1 VP1 is highlighted in magenta, while that of CV-A9 is shown as an orange oval. The integrin-binding site in HPeV-1 as determined previously by cryo-electron microscopy is encircled by a solid line. The location of the footprint of the integrin receptor on CV-A9 (EMD-5512) is encircled by a dashed line. The conserved RGD motif in FMDV (PDB code 1FOD) is shown as a light-blue oval. The positions of the icosahedral-symmetry axes are indicated by a pentagon (5-fold), triangles (3-fold), and an oval (2-fold).

The integrin α_V_β_6_ is a cellular receptor for HPeV-1 (24, 25). The integrin binds to an integrin recognition motif, RGD, in the C terminus of VP1. However, the RGD residues are not visible in the HPeV-1 electron density map, probably due to the flexibility of the loop that contains the sequence. The last structured residue of VP1 is Thr-216, which is 6 residues before the integrin-binding sequence (Fig. 5). Seitsonen et al. used cryo-electron microscopy to calculate a three-dimensional reconstruction of the HPeV-1–integrin complex (25). Rigid-body fitting of the HPeV-1 virion into the map of the complex enabled us to estimate the location of the RGD motif to be approximately in the middle between the icosahedral 5-fold and 3-fold axes immediately above the last structured residue of VP1 (Fig. 5; see Fig. S1 in the supplemental material). In contrast to HPeV-1, the integrin-binding site in foot and mouth disease virus (FMDV) is located above the core of subunit VP2 in the GH loop of VP1 (Fig. 5) (68). In coxackievirus A9 (CV-A9), the integrin-binding site is next to the icosahedral 5-fold axis (Fig. 5) (69). Similar to HPeV-1, the RGD residues are not visible in the CV-A9 crystal structure (70). Thus, the location of the RGD sequence in the flexible part of the capsid protein might be required for binding to the integrin receptor. The distinct integrin-binding sites in HPeV1, FMDV, and CV-A9 indicate a convergent evolution in which the different viruses independently acquired the ability to utilize the integrins as receptors for cell entry.

### HPeV-1 virion-antibody interactions.

**FIG 6:**
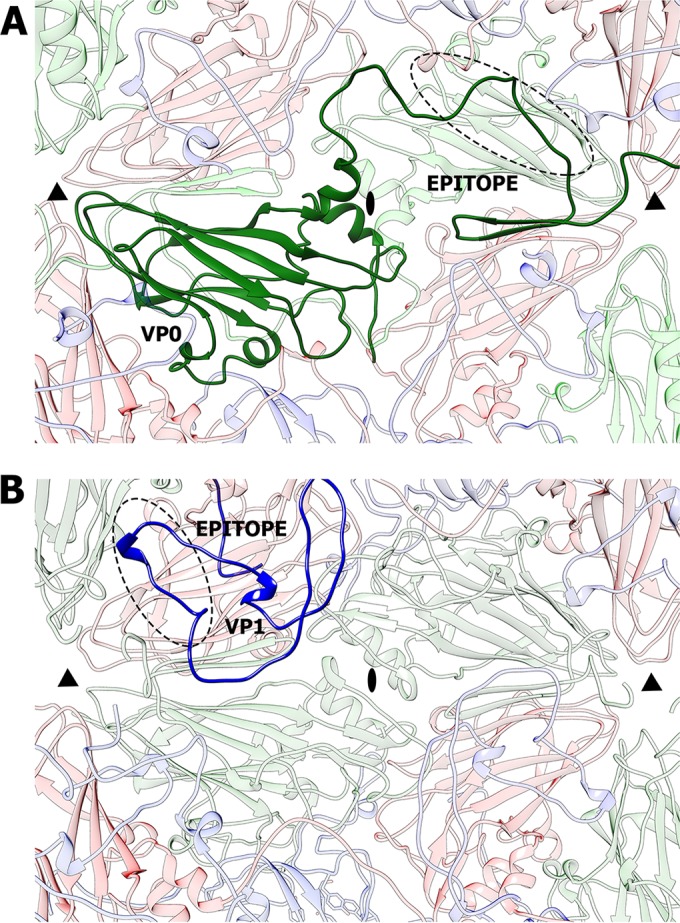
Comparison of internal immunogenic epitopes of HPeV-1 and poliovirus type 1 located at the interpentamer boundary. (A) Interior view of an HPeV-1 particle. A selected VP0 subunit is shown in dark green. Residues 82 to 94, targeted by the neutralizing antibodies, are highlighted with a dashed oval. (B) Interior view of a poliovirus type 1 particle. The N-terminal arm of a selected VP1 subunit is shown in dark blue. The immunogenic epitope, consisting of residues 33 to 54 of VP1, is highlighted with a dashed oval. The positions of the 2-fold and 3-fold icosahedral-symmetry axes are indicated by ovals and triangles, respectively.

An intravenous immunoglobulin infusion containing large amounts of HPeV-1-neutralizing antibodies proved to be an efficient treatment for parechovirus-induced cardiomyopathy in an infant (3). One of the strongest antigenic sites of HPeV-1 consists of residues 82 to 94 in the N-terminal arm of VP0, which form an extended loop connecting β3 and α2 on the inside of the capsid (Fig. 6A) (71, 72). A possible explanation for the high immunogenicity of a sequence that is inside the capsid is the dynamic motions of virions referred to as “capsid breathing.” Even residues that are located on the inside of the capsid can be temporarily exposed on the virion surface and may be accessible to antibodies. Similar targeting of internal peptides by antibodies has been described in other picornaviruses (73, 74). In poliovirus type 1, antibodies can bind to residues 34 to 53 of VP1 located on the interior of the particle (Fig. 6B) (73, 75). The buried antigenic sites of poliovirus type 1 and HPeV-1 are located at the interpentamer boundary (Fig. 6B). The immunological reactivity of the buried epitopes suggests that they might be temporarily exposed on the capsid surface without disrupting the integrity of the virion.

### Role of N termini of VP0 subunits in mediating interpentamer contacts within the HPeV-1 capsid.

**FIG 7:**
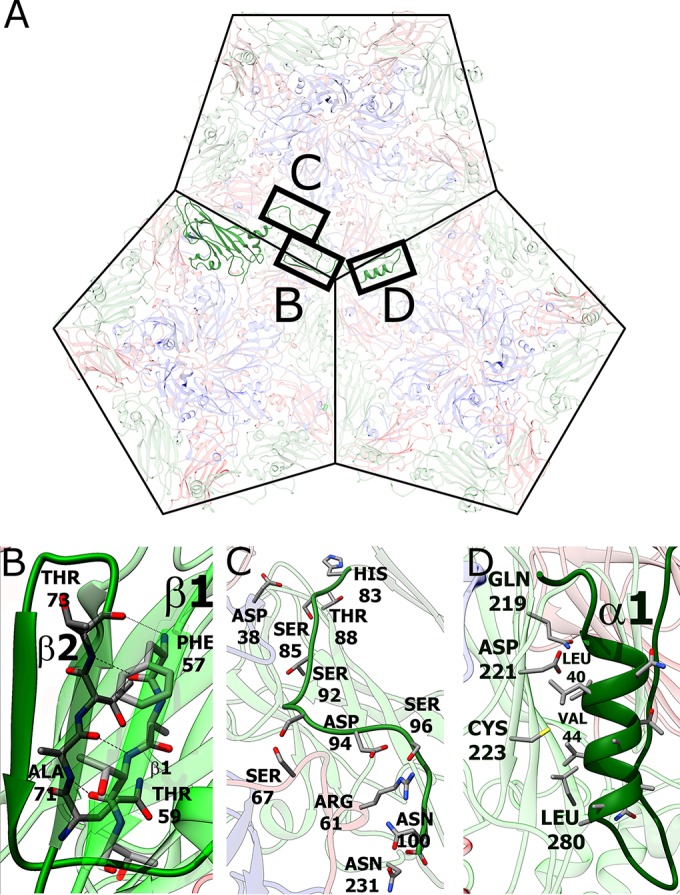
Role of the N-terminal arm of VP0 in interpentamer interactions. (A) The N-terminal arm of VP0 mediates contacts among three pentamers in the HPeV-1 virion. Shown is a view from inside the virion. (B) Interactions between antiparallel β-strands β1 and β2 of VP0 proteins from two adjacent pentamers. (C) Residues 83 to 100 of VP1 interact with residues from all three subunits, VP0, VP1, and VP3. (D) Interactions of α1 with βG of VP0 from another, neighboring pentamer.

The N-terminal part of HPeV-1 VP0 corresponds to the residues of VP4 and of the N terminus of VP2 in enteroviruses, according to the positions of the amino acids in the polyprotein precursors of the capsid proteins. However, the locations of the structured residues of the N terminus of VP0 in the HPeV-1 capsid are different from those of VP2 and VP4 in poliovirus type 1 and other enteroviruses while they are similar to those of HAV (Fig. 4). In HPeV-1, the structured N-terminal part of VP0 forms a tentacle-like extension that interacts with capsid proteins from two neighboring pentamers (Fig. 7A). Residues from the VP0 N terminus following helix α1 form stabilizing interactions with other parts of the capsid, including the following: (i) residues between β3 and α2 of VP0 form hydrogen bonds to Ser-67 and Arg-71 of VP3 and Asp-38 of VP1 subunits in the neighboring pentamer (Fig. 7B) and (ii) β1 of VP0 forms a two-stranded antiparallel β-sheet with β2 of VP0 in another adjacent pentamer (Fig. 7C). The N-terminal part of parechovirus VP0 might be homologous to VP4 of enteroviruses, which has to be released from the virion in order to ensure delivery of the virus genome across the endosomal membrane into the cytoplasm. Therefore, upon cell entry, the N terminus of VP0 might be required for interaction with the host cell membranes. Since the VP0 N termini are involved in interpentamer interactions, alteration of their structure leads to capsid disruption. Thus, the role of the VP0 N-terminal arm in mediating interactions among the pentamers within the HPeV-1 capsid might explain why the parechovirus capsids disassemble into pentamers after genome release *in vitro* (36). The first N-terminal residues of VP0 that are visible in the HPeV-1 electron density map form helix α1, which contains predominantly hydrophobic amino acids. The side chains of residues from helix α1 interact with the polar side chains of the residues from βG of subunit VP0 in the adjacent pentamer (Fig. 7D). The unfavorable nature of α1-βG interactions indicates that they are not required for capsid stability. The average B factor of α1 atoms is 51 Å^2^, while it is 40 Å^2^ for the whole capsid. The elevated B factor shows that the helix is more mobile and might be ready for exposure at the virion surface and interaction with membranes. However, it is possible that the part of the VP0 N terminus that is not visible in the structure (the first 30 residues) is responsible for interaction with the host membranes. In that case, the disruption of the HPeV-1 capsids into pentamers would not be required to ensure transfer of the ssRNA genome across the biological membrane.

### The putative role of the HPeV-1 genome in HPeV-1 virion stability and assembly.

The HPeV-1 virion has an inner volume of 6.9 × 10^6^ Å^3^ (Table 2). This is 20% smaller than the interior particle volumes of both poliovirus type 1 and rhinovirus type 16. However, the size of the genome of HPeV-1 is similar to that of poliovirus type 1, leading to a higher RNA density than in enteroviruses but similar to that in FMDV and HAV (Table 2). Furthermore, the internal surface of the HPeV-1 capsid around the 5-fold axes is more positively charged than the corresponding areas in other picornaviruses (see Fig. S2 in the supplemental material). This is probably required to neutralize the negative charge of the tightly packed RNA.

**FIG 8:**
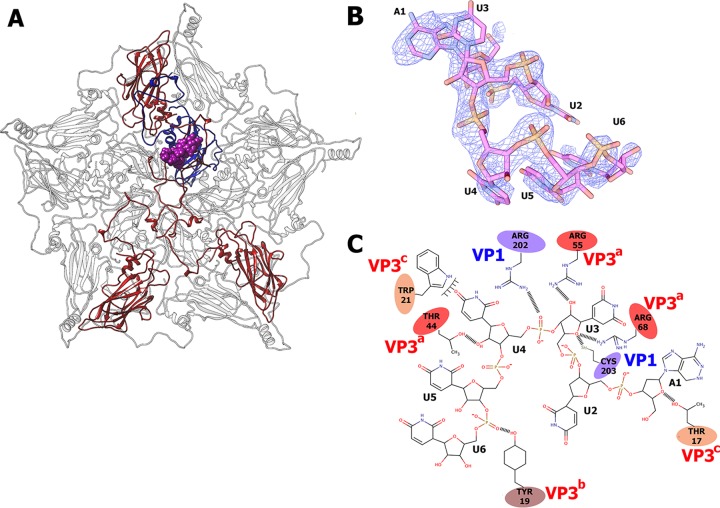
Interactions of HPeV-1 genomic RNA with the capsid. (A) Location of an RNA hexanucleotide (shown as a space-filling model in magenta) within the pentamer of capsid protein protomers as seen from inside the virion. Subunits in contact with RNA are shown in color (VP1, blue, and VP3s, red). (B) Electron density map of RNA hexanucleotide contoured at 2σ. (C) Two-dimensional representation of RNA-protein interactions. Hydrophobic interactions and hydrogen bonds are shown. VP3 subunits from different icosahedral asymmetric units are distinguished by the label colors and superscript a, b, and c.

An electron density corresponding to an RNA hexanucleotide associated with each icosahedral asymmetric unit is located close to the 5-fold icosahedral axis on the inside of the HPeV-1 capsid (Fig. 8). The HPeV-1 genome is a linear RNA molecule that forms unique interactions with the inner surface of the capsid. However, the RNA does not affect the packing of particles within the crystal or the measured diffraction data, and therefore, it contains information about the icosahedrally averaged RNA structure. Thus, the observed RNA density corresponds to an averaged nucleotide sequence. The shapes of the electron densities of the individual bases indicate that the first nucleotide is a purine, while the following 5 nucleotides are pyrimidines. The RNA was modeled as an adenosine followed by five uridines (Fig. 8B). The average B factor of the RNA is 71 Å^2^, while that of the protein part of the capsid is 40 Å^2^, indicating higher mobility of the RNA. A crystallographic refinement resulted in 94% occupancy of the RNA, showing that the RNA is bound to nearly all the available positions in the icosahedral capsid. Each hexanucleotide forms extensive interactions with one VP1 and three VP3 subunits belonging to different protomers from one pentamer (Fig. 8). Overall, the 60 copies of the ordered hexanucleotides represent 5% of the 7,500-nucleotide genome.

The HPeV-1 virion contains more ordered RNA than is seen in other picornaviruses, in which only 1 or 2 nucleotides are observed (27, 76, 77). For example, in enteroviruses, a nucleotide base makes stacking interactions with a conserved tryptophan residue of VP1 located close to the icosahedral 2-fold axis (76). However, the partly ordered genomic RNA has been previously observed in a bean pod mottle virus (BPMV) from the family Secoviridae of the order Picornavirales (78). In BPMV, the RNA interacts with the capsid proteins close to the icosahedral 3-fold axis, while in HPeV-1, the RNA binds close to the 5-fold axis (see Fig. S3 in the supplemental material). The differences in the RNA-protein interactions between the two viruses indicate that the two viruses might differ in the structures of their packaged genomes. It is likely that the genome organization in HPeV-1 virions is also different from that in enteroviruses, which may be due to the necessity to package the genome with a higher density. The complete conservation of the RNA binding residues among HPeVs, together with the nearly complete occupancy of the RNA, indicates that the binding might have a role in virion assembly and perhaps also in ensuring the selective packaging of HPeV genomes into capsids. Small molecules that interfere with the genomic-RNA–capsid protein interactions could therefore be developed into antiviral compounds preventing HPeV virion assembly.

## Supplementary Material

Supplemental material
