# Supplementary material for: The Structure of Human Parechovirus 1 Reveals an Association of the RNA Genome with the Capsid
Source: J Virol. 2016 Jan 15;90(3):1377–86. doi: 10.1128/JVI.02346-15 (PMC4719609; doi:10.1128/JVI.02346-15)
Supplement: Supplemental material [file supp_90_3_1377__index.html]

The Structure of Human Parechovirus 1 Reveals an Association of the RNA Genome with the Capsid — Supplemental material 

# The Structure of Human Parechovirus 1 Reveals an Association of the RNA Genome with the Capsid

## Supplemental material

- Supplemental file 1 -

  Fig. S1 (Fit of HPeV-1 virion crystal structure to cryo-EM reconstruction of HPeV-1–αvβ6 integrin complex.)

  Fig. S2 (Distributions of charge on the inner capsid surfaces of selected picornaviruses.)

  Fig. S3 (Ordered RNA segments in HPeV-1 and BPMV.)

  PDF, 4.6M
