# Supplementary material for: The Structure of Human Parechovirus 1 Reveals an Association of the RNA Genome with the Capsid
Source: J Virol. 2016 Jan 15;90(3):1377–86. doi: 10.1128/JVI.02346-15 (PMC4719609; doi:10.1128/JVI.02346-15)
Supplement: Supplemental material [file JVI.02346-15_zjv999091240so1.pdf]

Supplemental figure legends:

Figure S1. Fit of HPeV-1 virion crystal structure to cryo-EM reconstruction of HPeV-1 : αvβ6 integrin complex. **(A)** Rigid-body fitting of HPeV-1 virion (PDB code 4Z92) to the 8.7 Å cryo-EM reconstruction (EMDB code 1689) using fit-to-map function of UCSF Chimera. **(B)** Top view of surface of virion with interaction sites between virus and receptors highlighted by cyan spheres.

Figure S2. Comparison of distribution of charge on the inner capsid surface of selected picornaviruses. Blue indicates positive charge while red is negative. Pentamers of capsid protein protomers are shown. The scale of k_bT/e is used, where k_b is Boltzmann's constant, T is temperature, and e is the charge of an electron.

Figure S3. Comparison of ordered RNA segments in HPeV-1 and BPMV. Cartoon representation of the RNA-protein interactions in the icosahedral asymmetric unit of HPeV-1 **(A)** and BPMV (PDB code 1PGL). **(B)**. HPeV-1 VP0 is shown in green, VP1 in blue, VP3 in red, and RNA in magenta. Capsid proteins of BPMV are colored to correspond to the picornavirus convention. The subunit corresponding to VP1 is shown in blue. The other SBMV subunit contains two β-sandwich folds. Residues 1-184 corresponding to VP0 are shown in green and residues 185-370 corresponding to VP3 in red.

Figure S1

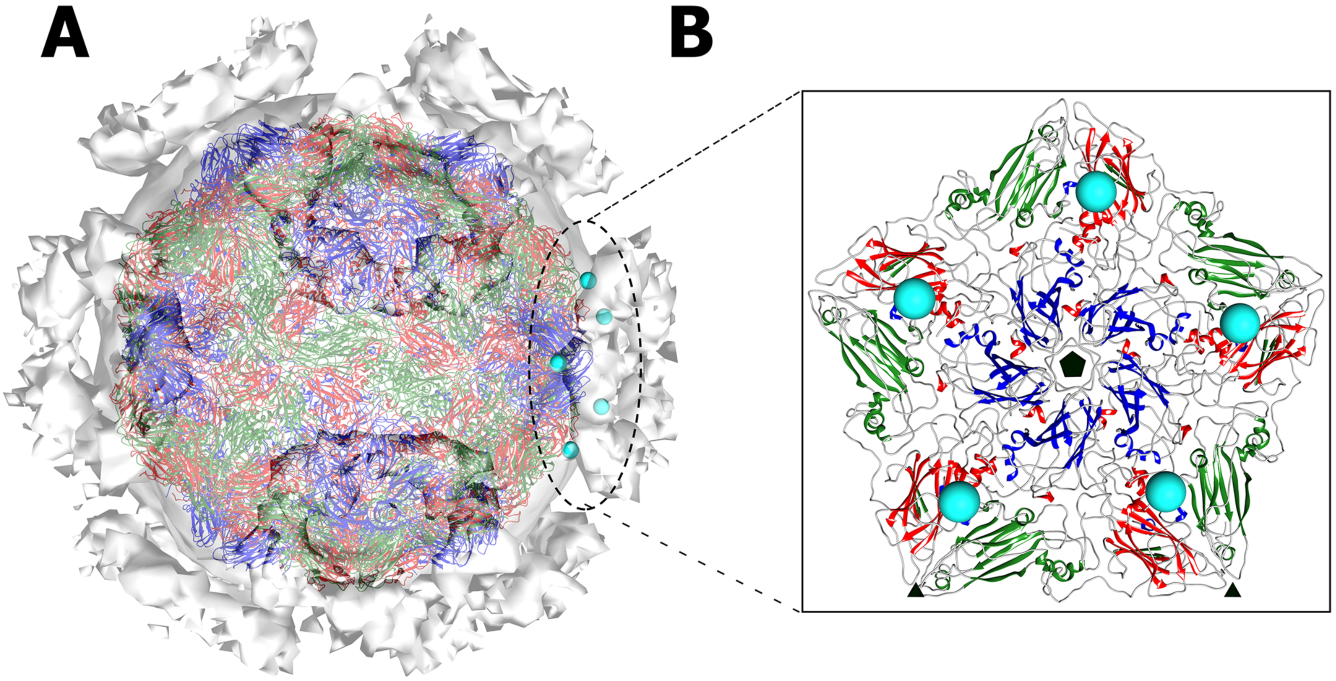

Figure S2

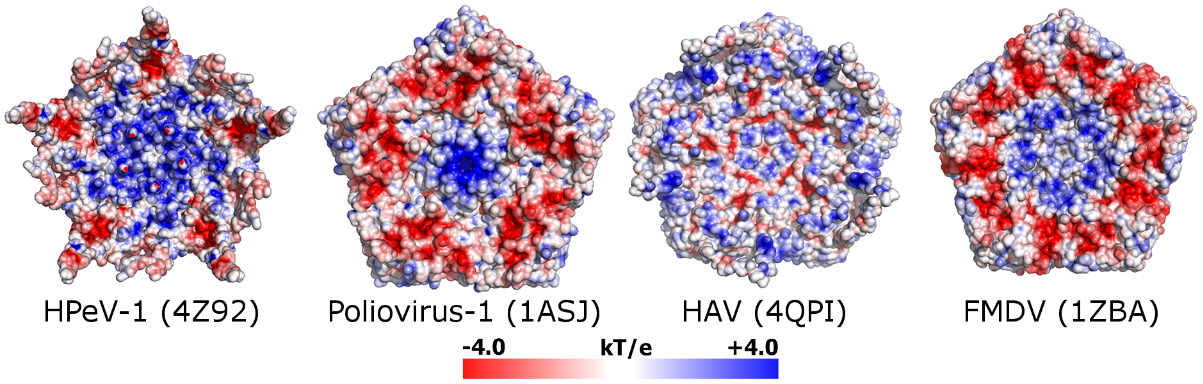

Figure S3

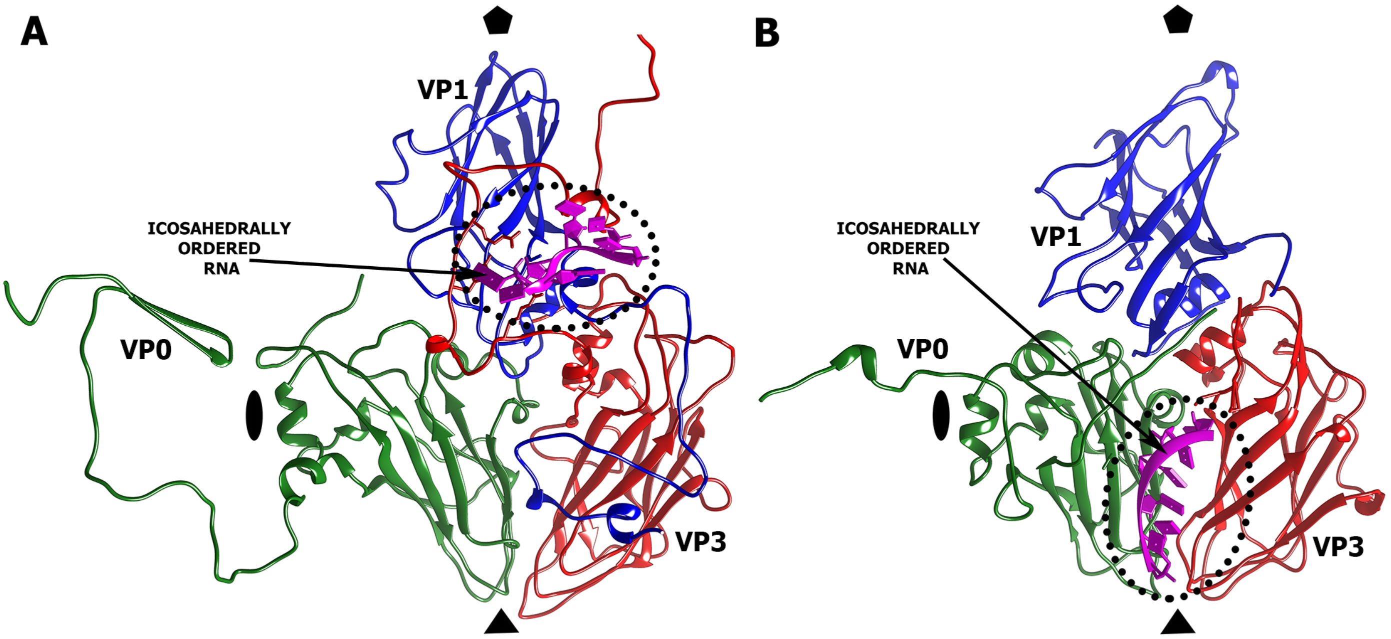
A
VP1
ICOSAHEDRALLY
ORDERED
RNA
VP0
VP3
B
ICOSAHEDRALLY
ORDERED
RNA
VP1
VP0
VP3
